# Brain-region–specific alterations of the trajectories of neuronal volume growth throughout the lifespan in autism

**DOI:** 10.1186/2051-5960-2-28

**Published:** 2014-03-10

**Authors:** Jerzy Wegiel, Michael Flory, Izabela Kuchna, Krzysztof Nowicki, Shuang Yong Ma, Humi Imaki, Jarek Wegiel, Ira L Cohen, Eric London, W Ted Brown, Thomas Wisniewski

**Affiliations:** 1 Department of Developmental Neurobiology, NYS Institute for Basic Research in Developmental Disabilities, 1050 Forest Hill Road, Staten Island, NY 10314, USA; 2 Department of Infant Development, NYS Institute for Basic Research in Developmental Disabilities, Staten Island, NY, USA; 3 Department of Psychology, NYS Institute for Basic Research in Developmental Disabilities, Staten Island, NY, USA; 4 Department of Human Genetics, NYS Institute for Basic Research in Developmental Disabilities, Staten Island, NY, USA; 5 Department of Psychiatry, Neurology and Pathology, New York University School of Medicine, New York, NY, USA

**Keywords:** Autism, Brain, Neuropathology, Neuronal growth, Subcortical structures, Cerebellum, Nucleator

## Abstract

Several morphometric studies have revealed smaller than normal neurons in the neocortex of autistic subjects. To test the hypothesis that abnormal neuronal growth is a marker of an autism-associated global encephalopathy, neuronal volumes were estimated in 16 brain regions, including various subcortical structures, Ammon’s horn, archicortex, cerebellum, and brainstem in 14 brains from individuals with autism 4 to 60 years of age and 14 age-matched control brains. This stereological study showed a significantly smaller volume of neuronal soma in 14 of 16 regions in the 4- to 8-year-old autistic brains than in the controls. Arbitrary classification revealed a very severe neuronal volume deficit in 14.3% of significantly altered structures, severe in 50%, moderate in 21.4%, and mild in 14.3% structures. This pattern suggests desynchronized neuronal growth in the interacting neuronal networks involved in the autistic phenotype. The comparative study of the autistic and control subject brains revealed that the number of structures with a significant volume deficit decreased from 14 in the 4- to 8-year-old autistic subjects to 4 in the 36- to 60-year-old. Neuronal volumes in 75% of the structures examined in the older adults with autism are comparable to neuronal volume in age-matched controls. This pattern suggests defects of neuronal growth in early childhood and delayed up-regulation of neuronal growth during adolescence and adulthood reducing neuron soma volume deficit in majority of examined regions. However, significant correction of neuron size but limited clinical improvements suggests that delayed correction does not restore functional deficits.

## Introduction

The clinical diagnosis of autism is currently based primarily on the observation of delayed or disrupted development of social and communication skills; of restricted, repetitive, and stereotypical patterns of behavior, interests, and activities; and having an onset prior to 3 years of age [1]. Autism does not have defining neuropathological diagnostic criteria, but smaller than normal neuron size has been reported in qualitative and quantitative studies. Bauman and Kemper demonstrated smaller neuron size and increased cell-packing density in the amygdala, medial septal nucleus, and cerebellar nuclei [2]. Casanova et al. [3] reported reduced size of neurons in the frontal and temporal cortex of autistic subjects. Reduced perikaryon volume of pyramidal neurons in the inferior frontal cortex in Brodmann areas 44 and 45 of autistic subjects [4], and in layers V and VI in the fusiform gyrus [5] support the hypothesis of the link between abnormal neuronal growth in some cortical regions and autism functional alterations.

However, all three diagnostic modalities of autism engage numerous subcortical structures. The amygdala is involved in processing of social information and emotional interpretation, as well as fear and anxiety [6-8]. The thalamus is implicated in language functions, attention, anxiety, and obsessive thinking [9,10]. Striatal functions indicate that the caudate and putamen can be involved in repetitive motor behaviors, compulsions, and rituals [11,12]. The brainstem and cerebellar deep nuclei integrate cerebellar contributions to motor function, language, cognition, and eye motion control [13,14]. Cholinergic neurons of the magnocellular basal complex modulate anxiety, arousal, and emotional and motor responses [15,16]. Although several postmortem morphometric studies have documented developmental alterations in the neocortex [4,5,17-19], cerebellum, and brainstem [20,21], the reported morphometric characteristics of the developmental alterations in subcortical structures are limited to the amygdala [22] and hippocampus [23]. Therefore, the first aim of this study was to test the hypothesis that autism is associated with global developmental alterations of neuronal growth including subcortical structures, hippocampus, archicortex, cerebellum, and brainstem.

Autism is viewed as a lifelong disorder whose clinical features change with development [24]. In nearly 50% of subjects later diagnosed with autism, the onset of functional alterations has been observed between 14 and 24 months, with all three diagnostic functional domains affected by age 24 months. Behavioral worsening in the second year paralleled by slowing of development [25] coincides with a rapid increase in head circumference at the age of 1 to 2 years [26-28]. However, a slower rate of brain growth between 2 and 4 years of age [28-30] results in only a 2% brain overgrowth in adults [31] or even a smaller brain size in comparison to control subjects [32]. The linkage between the deviation from the normal trajectory of brain growth and the severity of disease suggests a contribution of these changes to the clinical phenotype [26]. Modest improvements in communication [33-35], reciprocal social interactions [36], and restrictive, repetitive behaviors and interests [12,36] evident during the transition from childhood to adolescence, from adolescence to adulthood, and in adulthood define the behavioral trajectories of development and maturation of individuals with autism [35,37,38]. One may assume that the age-dependent alterations of neuronal growth in the subcortical structures, the cerebellum, and the brainstem are integral components of brain pathology that may contribute to different clinical manifestations of autism at different stages of life. In view of this, the second aim of this study was to characterize the differences between the trajectories of neuronal growth in (a) children, teenagers/young adults, and adults diagnosed with autism, and (b) age-matched control individuals.

The brain overgrowth seen in 2- to 4-year-old children with autism and associated with overgrowth of the frontal and temporal lobes and the amygdala, but not the occipital lobe, suggests desynchronized development of the brain subdivisions. The pathological acceleration of the growth of the brain regions involved in cognitive, social and emotional functions and in language development may contribute to the functional deficits observed in autism [28-30,39]. Neocortical studies in autism have revealed a 67% increase in the number of neurons in the prefrontal cortex [40], a 53% increase in the ratio between von Economo neurons and pyramidal neurons in the fronto-insular cortex [18], but a reduced number of neurons in the fusiform gyrus of autistic individuals [5]. These differences suggest regional disproportions and desynchronization of brain development in autism. Therefore, the third aim was of this study was to test the hypothesis that in subcortical structures, the hippocampus, the archicortex, the cerebellum, and brainstem, the severity of neuronal growth abnormalities is region-specific.

## Material and methods

Morphometric methods were applied to brains of 14 individuals with autism from 4 to 60 years of age, and 14 control subjects from 4 to 64 years of age. In the examined autistic cohort, there were 2.5 × more males than females (10 males and four females). The proportion between males and females was similar in the control group (nine males and five females).

In the first phase of this study, 39 brains were selected, including 21 from autistic subjects and 18 from controls. To reduce the risk of distorted results, strict clinical inclusion criteria and neuropathological exclusion criteria were applied. Two cases were excluded because they did not meet the Autism Diagnostic Interview-Revised (ADI-R) [1] criteria for a diagnosis of autism. In the autistic group, one brain was excluded because of severe postmortem autolysis distorting neuronal shape and size; three brains, because of hypoxic encephalopathy; and one, because of multiple microinfarcts. In the control group, four brains were excluded because of severe autolysis. These exclusions resulted in a reduction of the autistic group by seven cases (33%) and of the control group by four cases (22%).

To confirm the diagnosis of autism, the ADI-R was administered retrospectively (Table 1). The intellectual disability of eight subjects (61%) had been evaluated with the Wechsler Intelligence Scale for Children III and the Woodcock-Johnson Tests of Achievement-Revised, and ranged from mild to severe. Seizures were reported in seven of the 14 autistic subjects (50%). In five cases, death was seizure-related (36%). Self-injurious behavior was reported in six cases (43%), aggression in four (29%), hyperactivity in three (21%), obsessive compulsive disorder in two (14%), and depression and mania in a single case for each.

**Table 1:** Autism diagnosis, prevalence of seizures, and SUDEP

**Case number**	**Sex**	**Age (years)**	**Autism Diagnostic Interview- Revised (ADI-R)**			**Repetitive behavior (3)**	**Seizures age of onset and SUDEP**
			**Social deficits (10)**	**Communication deficits**			
				**Verbal (8)**	**Nonverbal (7)**		
1	M	4	14	NA	10	3	—
2	F	5	26	NA	11	4	—
3	M	7	29	NA	14	3	14 months
4	M	8	19	14	NA	4	—
5	F	11	22	14	NA	3	4.5 months, SUDEP
6	M	13	28	NA	12	3	2 years, SUDEP
7	F	17	15	16	NA	7	—
8	F	21	21	NA	11	7	5 years, SUDEP
9	M	22	28	NA	14	6	15 years, SUDEP
10	M	23	30	NA	14	8	23 years, SUDEP
11	M	36	23	NA	10	6	—
12	M	52					—
13	M	56	19	8	13	3	—
14	M	60	26	8	14	4	3 years

ADI-R cut-off score shown in parentheses.

SUDEP, unexpected and unexplained death that occurs in patients with known epilepsy.

### Tissue preservation and morphometric method**s**

The postmortem interval (PMI), corresponding to the period between death and autopsy, ranged from 6 to 28 h in the control group (16.7 h on average; standard deviation [SD] 6.6 h) and from 8 to 50 h in the autistic group (21.9 h on average; SD 11.4 h). The difference in the PMI between the two groups was not significant (Table 2). Selected structures were examined in 11 right hemispheres and 3 left hemispheres in the autistic and control groups.

**Table 2:** Tissue samples, demographics of autistic and control subjects, brain weight, and changes during processing

**Group**	**Case number**	**Sex**	**Age (years)**	**PMI (h)**	**Brain weight (g)**	**H**	**Fixation (days)**	**Dehydration (days)**	**Weight loss (%)**
A	1	M	4	30	1,280	R	4,560	28	49
A	2	F	5	13.2	1,390	R	1,568	28	52
A	3	M	7	25	1,610	R	330	37	55
A	4	M	8	22.2	1,570	R	196	36	45
A	5	F	11	12.9	1,460	L	308	34	52
A	6	M	13	8	1,470	L	75	33	39
A	7	F	17	25	1,580	L	470	36	51
A	8	F	21	50	1,108	R	136	35	43
A	9	M	22	25	1,375	R	1,034	39	38
A	10	M	23	14	1,610	R	533	45	60
A	11	M	36	24	1,480	R	721	37	44
A	12	M	52	27.6	1,625	R	1,650	39	40
A	13	M	56	3.3	1,570	R	692	38	52
A	14	M	60	26.5	1,210	R	398	39	38
Average				**21.9**	**1,453**		**905**	**36**	**47**
SD				**11.4**	**162**		**1,160**	**4**	**7**
p< (difference between autistic and control groups)				**ns**	**ns**		**ns**	**ns**	**ns**
C	1	F	4	17	1,530	R	126	41	49
C	2	F	4	21	1,222	R	233	30	43
C	3	M	7	12	1,240	R	130	41	51
C	4	F	8	20	1,222	R	650	36	51
C	5	M	14	20	1,464	R	1,067	38	44
C	6	F	15	9	1,250	R	372	41	49
C	7	F	20	9	1,340	R	245	37	52
C	8	M	23	6	1,520	R	95	45	41
C	9	M	29	13	1,514	R	89	41	49
C	10	M	32	24	1,364	R	460	37	42
C	11	M	48	24	1,412	L	215	38	39
C	12	M	51	18	1,450	L	1,819	20	26
C	13	M	52	13	1,430	L	158	47	48
C	14	M	64	28	1,250	R	52	37	51
Average				**16.7**	**1,372**		**408**	**38**	**45**
SD				**6.6**	**118**		**491**	**7**	**7**

*PMI* postmortem interval; *h* hours; *H* hemisphere; *R* right; *L* left; *SD* standard deviation.

Fixation: duration (in days) of fixation in 10% buffered formalin.

Dehydration: duration (in days) of dehydration in ethyl alcohol.

Weight loss: decrease of hemispheric brain sample weight during dehydration in ethyl alcohol.

The average weight of the brains in the autistic cohort (1,453 g) was not significantly different from that of the control group (1,372 g). The brain hemisphere with cerebellum and brainstem was fixed with 10% buffered formalin for an average of 408 days in the control group (range 52–1,819 days; SD 491 days). The average time of fixation in the autistic group was 905 days (range from 75 to 4,560 days, SD 1,160 days). The brains were dehydrated in a series of ascending concentrations of ethyl alcohol. The average time of dehydration was 36 and 38 days in the autistic and control group, respectively. Dehydration was associated with reduction of brain hemisphere weight by 47% (SD 7%) on average in the autistic group and by 45% (SD 7%) in the control group. Brain hemispheres were embedded in 8% celloidin [41]. Serial 200-μm-thick sections were stained with cresyl violet and mounted with Acrytol.

Autistic and control subjects were very precisely age matched. There was no significant difference between the autistic and the control group in the PMI, autopsy brain weight, fixation time, dehydration time, or brain weight loss during dehydration. Therefore tissue preservation and processing impact on age-related changes is expected to be comparable in both cohorts and to affect estimates of neuron volume in a similar way. Moreover, robust statistical methods were applied to minimize any distortion of results due to outlying cases.

The neuropathological examination was carried out (a) to identify the type, distribution, and severity of qualitative developmental changes and (b) to eliminate brain samples affected by either pathology not related to autism, changes associated with mechanisms of death, or postmortem autolytic tissue degradation. On average, 120 cresyl violet–stained serial hemispheric sections were examined per case in blinded fashion with regard to diagnosis. The results of our study of the developmental abnormalities in these cohorts were previously reported [42]. In summary, the neuropathological evaluation revealed a broad spectrum of focal developmental alterations in the 13 examined autistic brains, including (a) subependymal nodular dysplasia, (b) subcortical and periventricular heterotopias, and (c) dysplastic changes in the neocortex, archicortex, dentate gyrus, Ammon’s horn, and cerebellum. The pathology detected in 92% of the autistic brains reflects focal modifications of neurogenesis, migration, and alterations of the cytoarchitecture. The absence of these changes in the control brains indicated that the detected alterations were autism-associated.

The morphometric measurements of the material being analyzed were performed without knowledge of the subject’s age, gender, clinical diagnosis, or neuropathological status. The volume of neurons was estimated in 16 brain structures or neuronal populations, including the amygdala, entorhinal cortex, Ammon’s horn, claustrum, caudate nucleus, putamen, globus pallidus, nucleus accumbens, thalamus and two parts (magnocellular and parvocellular) of the lateral geniculate body (LGB), substantia nigra, the magnocellular basal complex (MBC), including the acetylcholinergic system; Purkinje cells and dentate nucleus in the cerebellum, and the inferior olive in the brainstem.

Neuronal morphometry was performed using a workstation consisting of an Axiophot II (Carl Zeiss, Goettingen, Germany) light microscope with Plan Apo objectives 1.25× (numerical aperture, N.A., 0.15); 2.5× (N.A. 0.075), 40× (N.A. 0.75), and 63× (N.A. 0.9), a specimen stage with a three-axis, computer-controlled stepping motor system (Ludl Electronics; Hawthorne, NY, USA), a CCD color video camera (CX9000 MicroBrightfield Bioscience, Inc., Williston, VT, USA), and stereology software (Stereo Investigator and Nucleator, MicroBrightField Bioscience, Inc.). The volume of neurons was estimated with the nucleator method [43] using the MicroBrightfield software (Nucleator).

### Nucleator

To eliminate bias related to sectioning defects, 5-μm top and bottom guard zones were applied. To preserve the same standard of evaluation of neurons of different sizes and shapes, five rays were used in cellular measurements in all 16 regions of interest. The center of the nucleator was laid down in the nucleolus. The observer first clicked on the nucleolus and then at the five points within the focal plane where five systematic randomly rotated radii intersected with the cell border. In large neurons, radii aligned with the long axis of thick dendrites required determination of the border between cell soma and processes. For the nucleator, an abrupt reduction of the intensity of cytoplasmic staining at the base of weakly stained processes was considered to be the neuron-soma border. The soma volume was estimated from the lengths of radial cellular segments.

### Neuron identification for nucleator

The neurons examined represented a broad spectrum of differences in size from the largest Purkinje cells in the cerebellum to the smallest neurons in the nucleus accumbens. In general, the neurons were distinguished from the glial cells using histological criteria provided by cresyl violet staining. The largest neurons are characterized with a low contribution of the nucleus volume to the cell body volume, whereas in smaller neurons the contribution of nucleus to cell body volume is higher. The examined neurons were larger than the glial cells, and had brain region–specific spatial orientations in examined brain structures, layers, sectors, and nuclei. Intense staining of nuclear chromatin in the small round nuclei in oligodendrocytes; small, often elongated nuclei in the microglial cells; and a round pale nucleus with a small amount of nuclear chromatin and lack of nucleolus in the astrocytes, distinguished the glial cells from neurons with a prominent nucleus and nucleolus and a distinct marginal and central chromatin.

### Sampling scheme

The parameters and procedures that were applied to estimate the volume of the neuronal soma in the 16 brain structures are presented in (Additional file 1: Table S1). An optical fractionator systemic random sampling scheme was applied (Stereo Investigator, MicroBrightField Inc.). At least four equidistant serial sections [44] were used for neuronal measures in the anatomical subdivisions of the amygdala (total 12 sections), substantia nigra (total 9 sections), Ammon’s horn sectors CA1–CA4) (total 14 sections), claustrum (total 9 sections), and magnocellular basal complex (total 9 sections). In the large striatal subdivisions (caudate nucleus, putamen, and globus pallidus) with a relatively uniform cytoarchitecture, four sections were examined. Examination of the relatively large number of neurons (213/ROI/case, on average) resulted in a less than 0.01 Scheaffer coefficient of error (CE). The coefficient of error is essentially a measure of the uncertainty of the population estimate. The number of neurons measured in individual region has been controlled to reach a CE close to the required 0.05 (MicroBrightField, Inc.). Scheaffer coefficient of error decreases with an increasing number of measured neurons, counting frames per section and number of sections. The grid size and the virtual counting space were designed for each brain structure individually to adjust to the size and shape of the region of interest and to reduce the variation, as reflected in the SD and the CE. Very heterogeneous structures were oversampled (more sections, denser grid, more counting frames and more neurons measured) to achieve acceptable precision. The mean number of virtual counting spaces ranged from 59 for very small magnocellular basal complex (CE, 0.005), to 664 for Purkinje cells, with irregular distribution and low numerical density (CE, 0.002).

The results of the examination of the cytoarchitectonic subdivisions of the amygdala, entorhinal cortex, Ammon’s horn, and substantia nigra are presented as a mean value for the entire structure.

### Evaluation of measurement reliability

As random differences in the axes upon which measurements are taken are introduced by the Stereo Investigator protocol to avoid a measurement bias, per-neuron reliability cannot be meaningfully computed for each observer (or among observers). Consistency of each pathologist’s measurements was assessed by comparing repeated measurements of structures by the same rater. Interrater reliability was assessed as an intraclass correlation coefficient using data from a sample of five structures, each of which was examined by all four observers (raters). The raters were considered to be a random sample of all possible raters, so a random effects model was employed. This corresponds to ICC(2,1) proposed by Shrout and Fleiss [45], though the computed ICC is the same if the raters are assumed to comprise all possible raters, that is, if a mixed model assumption is employed, equivalent to ICC(3,1). As observers’ measurements are not in practice pooled, the reliability of their absolute agreement was assessed (rather than the more liberal standard of their consistency).

The evaluation of intrarater consistency revealed that differences in observers’ repeated measurements of a single structure ranged from 0.1% to 6.8%. The mean absolute value of the difference between the two ratings was 2.3%. Random differences in the axes upon which measurements were taken, introduced by the Stereo Investigator protocol to avoid a measurement bias, constitute a small component of these differences. Interrater reliability for five structures, each measured by all four observers, was very strong, with an intraclass correlation coefficient of .961 (95% C.I. .835, .995).

### Anatomical boundaries of examined brain structures and their cytoarchitectonic subdivisions

In this study, five components of the basal ganglia, including the telencephalic striatum (the caudate nucleus, putamen, nucleus accumbens), the diencephalic pallidum (external and internal globus pallidus), the mesencephalic substantia nigra, and the magnocellular basal complex, including the nucleus basalis of Meynert, were examined [46]. They are an integral part of the cortico-subcortical circuits involved in the programming and execution of movements, and they provide the necessary timing (ramp signals) for smooth guided movements. Abnormal movements and changes in muscle tone (hyper- or hypotonia) are prominent signs of basal ganglia disorders [46,47].

The caudate nucleus and putamen represent one cellular mass (striatum) partially separated by a thick internal capsule. The putamen extends from the external capsule to the external medullary lamina of the pallidum. In the ventral striatum, the nucleus accumbens was examined. The n. accumbens is located underneath the rostroventral aspect of the internal capsule. The border between the nucleus accumbens and dorsal striatum (caudate nucleus and putamen) was arbitrarily defined by a line perpendicular to the midline of the internal capsule at its lower end [48]. The study concentrated on the striatal small neurons, whereas a few sparse large neurons [49] were not included in this neuronal size analysis. The globus pallidus is separated from the putamen by the external medullary lamina, whereas the medial medullary lamina divides the globus pallidus into the external and internal pallidum [46]. Neurons in both parts of the pallidum were examined.

In the substantia nigra, the pars compacta was distinguished from the underlying pars reticulata by the accumulation of melanin in neuronal soma, and the nucleator examination was limited to melanin-positive neurons [50]. To identify the three major subdivisions of the pars compacta, Fearnly and Lees’ [51] classification of neuronal nuclei into the dorsal and ventral tier was used. The bundle of the putaminonigral pathway was used as a marker separating the pars lateralis from the ventral tier.

In a series of CV-stained sections, four subdivisions of the magnocellular basal complex, including the most prominent nucleus basalis of Meynert [52], were identified. Four subdivisions (Ch1-4) were delineated using Vogels [53] mapping. The small Ch1 magnocellular group is located in the medial septum, whereas a substantial population of magnocellular neurons is found in the Ch2 nucleus of the vertical limb of the diagonal band of Broca. Ch2 neurons are continuous with a sparser population of large neurons that are located along the medio-ventral surface of the hemisphere in the nucleus of the horizontal limb of the diagonal band (Ch3). At the preoptic area, the magnocellular group of the basal nucleus of Meynert (Ch4 group) [54] was examined. In the amygdala, the boundaries of the lateral, basal, accessory basal and central nuclei were identified on CV-stained sections using cytoarchitectonic criteria described in detail by Schumann and Amaral [22].

The mediodorsal surface of the thalamus is limited by the wall of the lateral ventricle, whereas the lateral and dorsal border of the thalamus is accentuated by the reticular thalamic nucleus that separates the thalamus from the posterior limb of the internal capsule. To obtain global characteristics of the thalamus, which consists of more than 50 nuclei with often poorly defined borders, the entire thalamus was randomly sampled using equidistant sections.

A thin ribbon of claustrum gray matter is separated from the insula by the extreme capsule and from the putamen by the external capsule [49]. Two major anatomical subdivisions including the claustrum (compact insular claustrum and ventral claustrum), and the prepiriform claustrum were examined. Poorly defined and diffuse subregions such as preamygdalar claustrum [55] were excluded from this study.

Purkinje cells were identified on the basis of their distribution along the border between the molecular and granular layer, their large soma size, and their apical dendrite spatial orientation. The study of deep cerebellar nuclei was limited to neurons within the dentate nucleus to eliminate random contributions of neurons of the small and medially located fastigial, globose, and emboliform nuclei.

The study of the inferior olive, divided into a principal nucleus, dorsal and medial accessory olives, dorsal cap of Kooy, ventrolateral outgrowth, and nucleus *β*[56], was limited to the largest principal nucleus.

The entorhinal cortex (EC, Brodmann area 28) spreads over the gyrus ambiens and the anterior portion of the hippocampal gyrus. The anterior limit of the EC starts about 5 mm rostrally to the amygdaloid complex. The EC ends anterior to the rostral pole of the lateral geniculate body. Rostrally, the medial portion of the EC borders with the preamygdaloid cortex in the area marked by the sulcus semiannularis. Caudally, the median portion of the EC borders with the subicular complex. The lateral border is distinguished as the border with Brodmann area 35 that lacks a distinct layer IV [57]. The transentorhinal zone [58] was not included in this study.

Neurons were measured within four layers of the EC. Layer II consists of islands of relatively large modified pyramidal and stellate neurons separated by an acellular gap from layer III [58]. A broad layer III is built up of medium-sized pyramidal neurons separated from layer V by an acellular lamina dissecans labeled as layer IV. Layer V consists of the sublayer Va with large pyramidal neurons, Vb with smaller and more loosely arranged neurons, and sublayer Vc with relatively few dispersed neurons. Layer VI is built up of the smallest neurons, and the border of this layer in the rostral portion is characterized by a decreasing gradient of neuronal density, whereas in the caudal portion the border is sharp [57].

Ammon’s horn neurons, in the pyramidal layer sectors CA1–4, were examined. The CA1 sector pyramidal neuronal layer extends from the subiculum to the CA2 sector as a thick band of relatively small pyramidal neurons. The border between the CA1/CA2 sector and CA2/CA3 sector is accentuated by a compact arrangement of large pyramidal neurons in CA2. Moreover, the pyramidal layer is narrowest in CA2. The size of neurons in the CA3 and 4 is similar, but parallel arrangement of neurons in the CA3 sector distinguishes CA3 from the hilus neurons (identified also as CA4 sector) [59,60]. The mapping of sectors on serial sections representing the entire rostro-caudal extension was based on the Amaral and Insausti [57] classification, but with the CA4 identification as a separate subdivision.

A six-layered lateral geniculate nucleus (LGN) borders at the base (hilus) [61] with the transverse fissure, whereas the posterior limb of the internal capsule separates the LGN from the lateral wall of the thalamus. The major portion of the LGN is present on the level of the border between the head and body of the hippocampus. Neurons were measured separately in two major ventral laminae (1 and 2; magnocellular LGB) and in the parvocellular laminae (3 to 6; parvocellular LGB), with neurons approximately half the size of neurons in the magnocellular laminae.

Tissue and medical records were handled in accordance with the NIH Guide for the use of human tissue. The research project and protocols were approved by the Institutional Review Board of the New York State Institute for Basic Research in Developmental Disabilities (IBR). The tissue was obtained from the Harvard Brain Tissue Resource Center, the Brain and Tissue Bank for Developmental Disorders of the National Institute of Child Health and Human Development at the University of Maryland, the Brain Bank at IBR, Staten Island, NY, and the Mount Sinai Medical School, NY. Tissue samples were coded with the number assigned by the brain banks, and this ID was the only identifier of the tissue, MRI scans, and clinical records. The Autism Tissue Program (Autism Speaks, Princeton, NJ) provided access to brain tissue samples and to the database with coded and anonymous characteristics of the autistic subjects, including the subject’s age at time of death, clinical diagnosis, results of application of the ADI-R, cause of death, PMI, and fixation time.

### Statistical analysis

This study analyzed the effect that autism has on neuronal soma, with age-matched controls as a comparison group. Data have been post-stratified to adjust for the differing numbers of neurons sampled per individual so as to weight each individual equally. Any data points lying more than 1.5 times the interquartile range below the 25th percentile or an equal amount above the 75th percentile for each structure or subdivision were considered outliers and omitted from analyses. Removed defective records accounted for 0.3% of all records.

Analyses were carried out separately for three groups of cases: 4 to 8 years, 11 to 23 years, and 36 to 60 years of age. Three groups of controls were selected to provide age-matching at the group level. The age ranges for control groups were 4 to 8 years, 14 to 23 years, and 29 to 64 years of age. Comparisons were carried out across 16 regions. Adjustment for multiple comparisons was made using the Benjamini-Hochberg method [62] to maintain a False Discovery Rate (FDR) of 0.05. Accordingly, p values of 0.027 or less were considered statistically significant. Analyses were conducted using versions 11.1 and 12.1 of the Stata statistical package [63,64].

Adjusted mean volumes and standard errors were computed using the multilevel sampling survey data procedures of the Stata package. Preliminary analyses regressed neuronal size, normalized by expressing each neuron’s size as a proportion of the size of the mean control neuronal size for that structure, on PMI (in hours), fixation time (in days), brain weight (in grams), brain weight loss during processing and dehydration (as a percentage), duration of dehydration (in days), autism status and log age (in years). This analysis resulted in the detection of significant effects for each of these factors. Age and these potential confounders of autism’s effect on neuronal size accounted collectively for 1.09% of the variance in size. Autism status, when entered into the model, raised the explained variance to 1.91%. As autism status univariately explained 1.45% of variance, all potential confounders considered together diminished the variance explained by the autistic status by approximately 43%, while autism remained by a large margin the strongest predictor in the model.

Further analyses of the potential effects of confounders, including analyses of the effect of a history of seizures and of sudden and unexplained death in patients with known epilepsy, were performed using the Stata svy:regress command, with potential confounders and autistic status (or age group) entered as predictors of neuronal or soma volume.

The statistical significance of the differences in mean neuronal volumes between autistic and control brains and between pairs of age ranges was computed in general linear mixed models adjusted for autocorrelation of neurons within each brain using the xtmixed procedure in version 12 of the Stata statistical package. This method takes into account the variance between neurons while assessing the differences between brains of autistic and control individuals. Exploratory analyses computing Generalized Estimating Equations (GEEs) using the Stata xtgee procedure yielded nearly identical results. Analyses were controlled for post-mortem interval (PMI, log-transformed), days of dehydration, and weight loss. Fixation time was also examined as a potential confounder but proved both redundant and highly correlated with the other control variables and often resulted in non-converging models. In a small number of models PMI was also removed to allow stable estimation. With the sole exception of the dentate nucleus in older subjects, controlling for seizures or sudden unexplained death in epilepsy (SUDEP) in analyses did not affect the significance of the difference in cell soma volume between autistic and control subjects.

## Results

**Figure 1:**
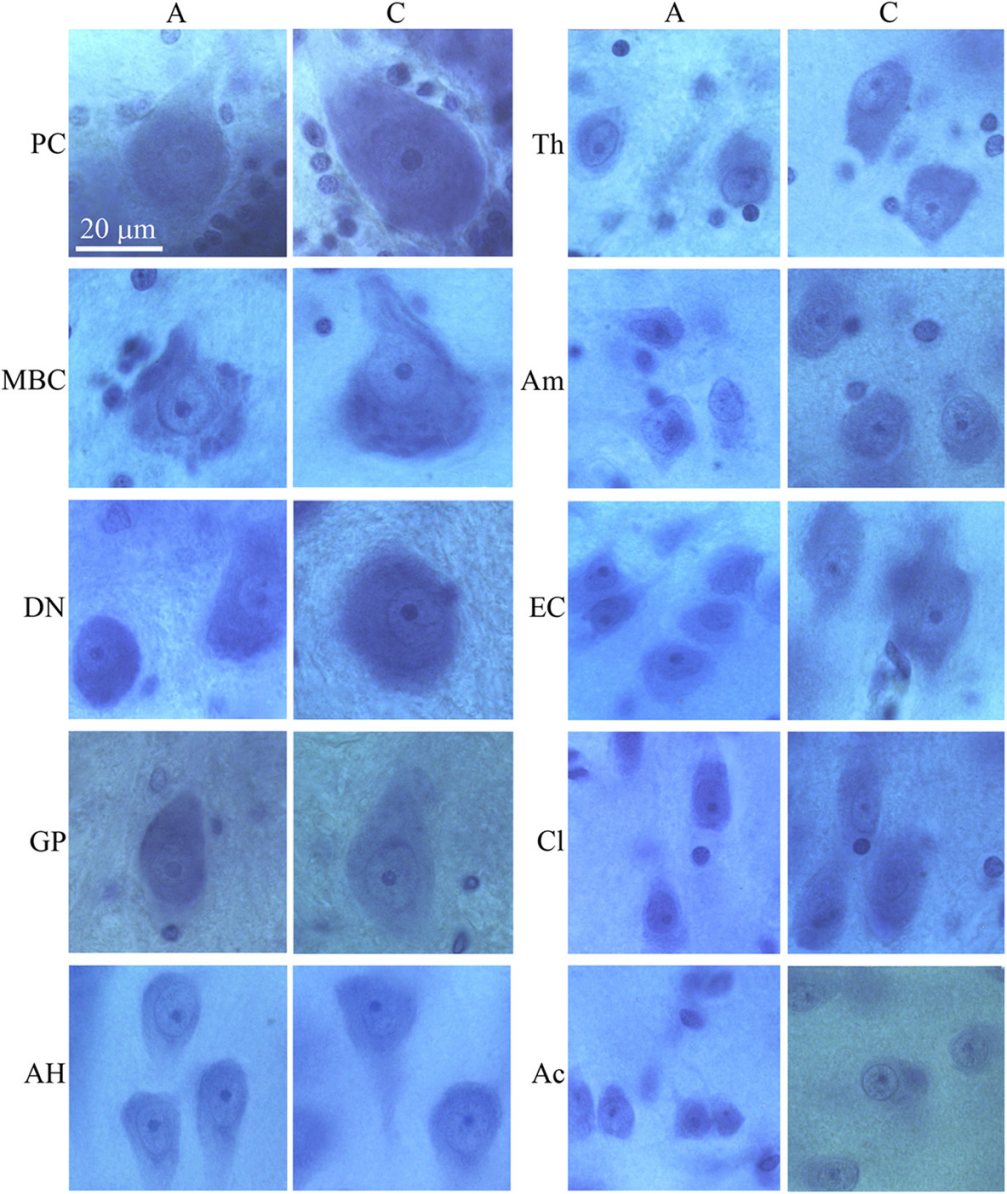
**Smaller size of neurons of autistic subject 8 years of age (A) in comparison to age-matched control subject (C).** Neurons shown in order from the largest to the smallest: Purkinje cells (PC), neurons in the magnocellular basal complex (MBC), dentate nucleus (DN), globus pallidus (GP), Ammons horn (AH), thalamus (Th), amygdala (Am), in the island in the second layer of the entorhinal cortex (EC), claustrum (Cl) and nucleus accumbens (Ac). Calibration bar, 20 μm.

**Figure 2:**
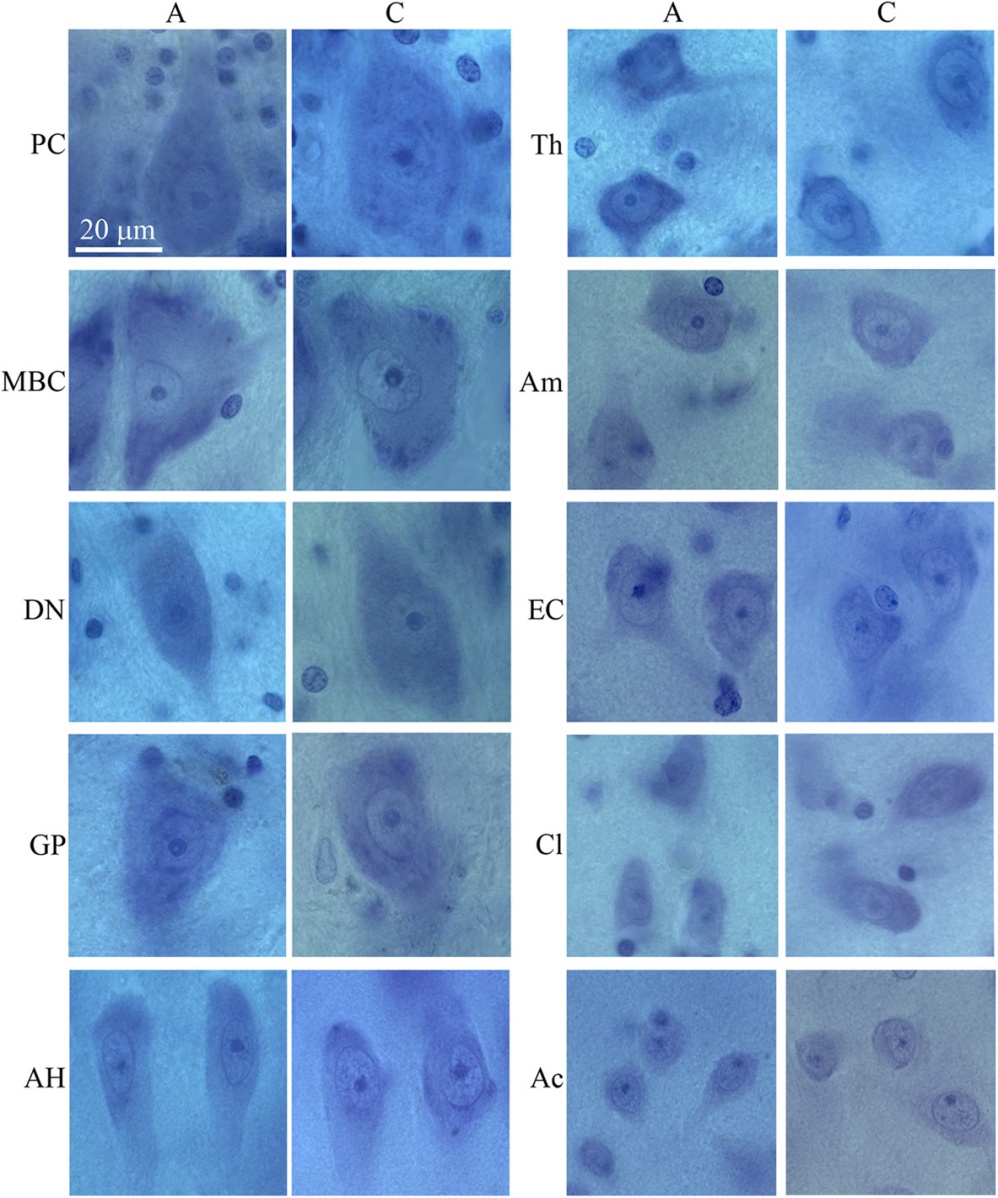
**Panel demonstrates the difference between neuronal size in 10 regions in 23 year of age autistic and 23 year of age control male.** At this age only Purkinje cells (PC), neurons in the dentate nucleus (DN), and claustrum (Cl) are smaller in autistic than in control subject. Neuronal volume differences are undetectable in the magnocellular basal complex (MBC), globus pallidus (GP), Ammons horn (AH), Thalamus (Th), amygdala (Am), entorhinal cortex (EC) and nucleus Accumbens (Ac). Calibration bar, 20 μm.

Examination of the CV-stained sections revealed brain region-specific differences in neuron size in control and autistic subjects. Neurons in the majority of the examined regions in 4- to 8-year-old autistic subjects were smaller compared to those in age-matched controls (Figure 1). However, in older subjects, the difference was less marked or was undetectable in the majority of examined regions (Figure 2).

### Region-specific neuronal volume deficit in autistic 4- to 8- year-old subjects compared to age-matched control subjects

**Figure 3:**
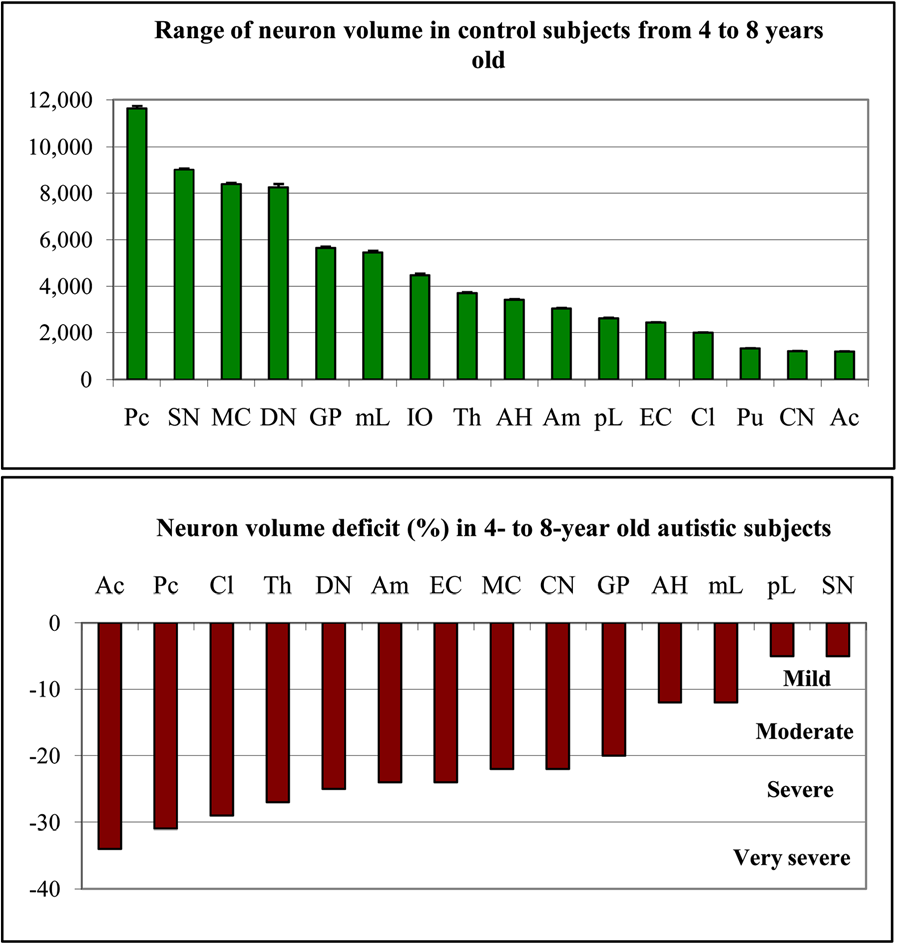
**The graph illustrate regional differences of neuron volume and neuron volume deficit in autism.** Upper graph shows the range of the mean volume of neuronal soma in 16 examined brain structures from the largest Purkinje cells (PC) to the smallest neurons in the nucleus accumbens (Ac) in from 4- to 8-year old control subjects. In 14 examined structures, the neuronal soma volume was significantly less (p < 0.05) than in age-matched control subjects, but the volume deficit (control, 100%) varied in examined regions and ranged from mild (< 10%), moderate (11–20%), severe (21–30%) to very severe (> 30%). Whiskers, SE. Symbols: Purkinje cells (Pc), substantia nigra (SN), magnocellular basal complex (MC), dentate nucleus (DN), globus pallidus (GP), magnocellular LGB (mL), thalamus (Th), Ammons horn (AH), amygdala (Am), parvocellular LGB (pL), entorhinal cortex (EC), claustrum (Cl), caudate nucleus (CN), and nucleus accumbens (Ac). The deficit in the putamen (Pu) and in the inferior olive (IO) was not significant.

The 16 brain regions examined represent a broad spectrum of brain structure– and neuron type–specific differences in neuronal soma size (Figure 3). In the 4- to 8-year-old control subjects, the range of volumes of the examined neurons extended from very large neurons such as Purkinje cells (11,635 μm^3^), dopaminergic neurons in the substantia nigra (9,008 μm^3^), and neurons in the magnocellular basal complex (8,385 μm^3^) to very small neurons in the striatum, including the putamen (1,316 μm^3^), caudate nucleus (1,199 μm^3^), and nucleus accumbens (1,181 μm^3^) (Additional file 1: Table S2).

The application of the nucleator to estimate the difference between neuronal soma volumes in four autistic and four control subjects 4 to 8 year of age revealed a significantly smaller volume of neurons in 14 of 16 brain regions examined (FDR < 0.05) in the autistic subjects. However, the reduced volumes of the neuronal soma varied within the examined regions over a very broad range from 5% to 34%. An arbitrary categorization of the severity of the developmental deficits identified two brain regions with a very severe (> 30%) neuronal volume deficit, seven regions with a severe (22%–29%) deficit, three regions with a moderate (12%–20%) deficit, and two regions with a mild (5%) volume deficit (Figure 3). A very severe volume deficit was found for the nucleus accumbens (−34%) and for Purkinje cells (−31%). A severe volume deficit was detected for the claustrum (−29%), thalamus (−27%), dentate nucleus (−25%), amygdala and entorhinal cortex (−24%), magnocellular basal complex and caudate nucleus (−22%). A moderate volume deficit was detected in the globus pallidus (−20%), Ammon’s horn and magnocellular lateral geniculate body (LGB) (−12%). A mild, but still statistically significant, deficit was found for the parvocellular LGB (−5%) and the substantia nigra (−5%). Deficits in the putamen and in the inferior olive were found not significant.

**Figure 4:**
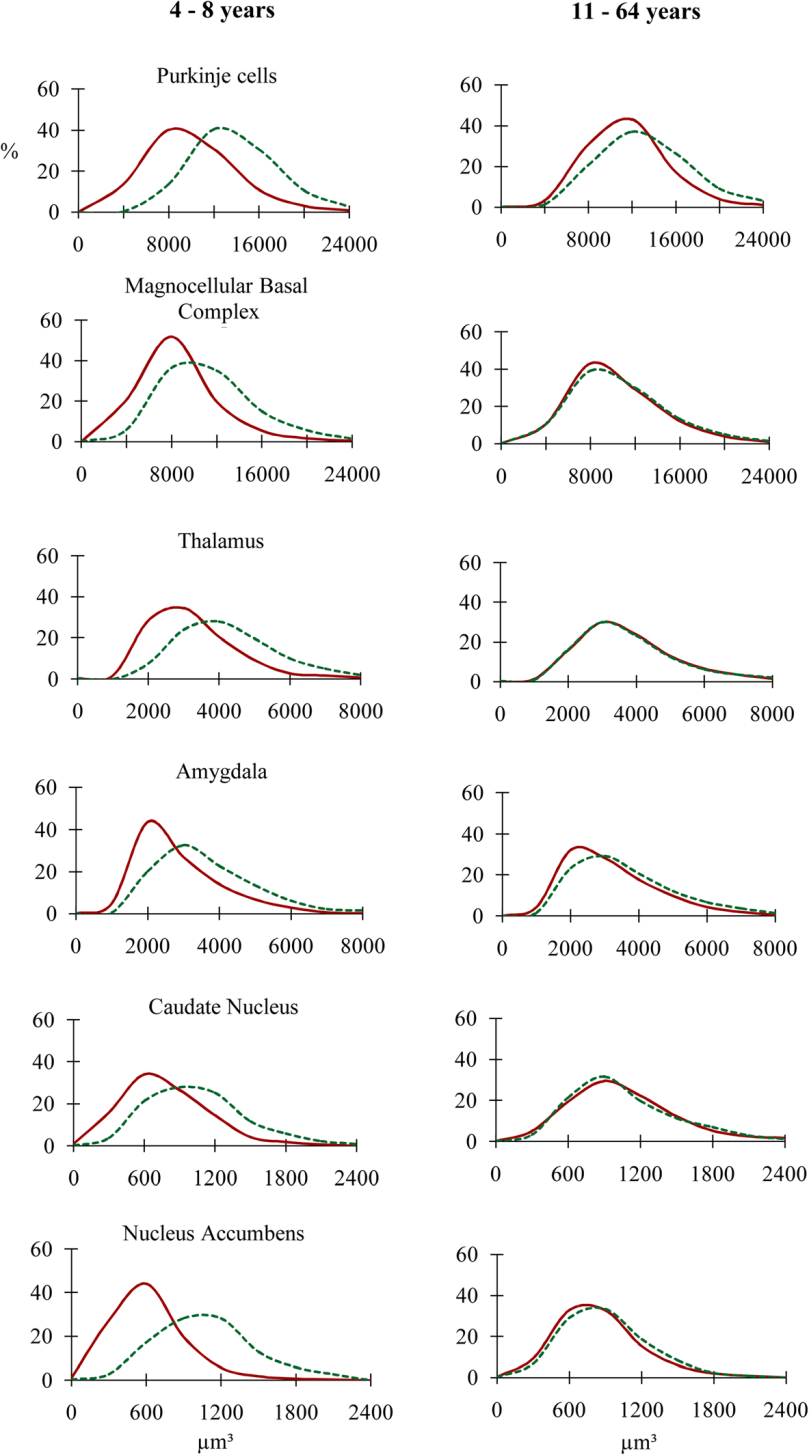
**Neuronal soma volume distribution in autistic (red line) and control (green line) subjects in brain structures with the largest neurons (Purkinje cells and neurons in the magnocellular basal complex), moderate size neurons (thalamus and amygdala), and small neurons (caudate nucleus and nucleus accumbens).** Graphs show a significant shift towards small size in 4- to 8-year-old autistic subjects in all three neuronal size categories and a rather uniform reduction of differences in volume distribution in 11- to 60-year-old autistic subjects.

Figure 4 illustrates the large contribution of smaller neurons in the autistic 4- to 8-year-old subjects in comparison to the controls and the partial or almost complete overlap of the distribution curves in the 11- to 64-year-old subjects. In general, this pattern was similar for large neurons (Purkinje cells and neurons in the magnocellular basal complex), medium-size neurons (thalamus, and amygdala); and small neurons (caudate nucleus and nucleus accumbens).

### Region-specific trajectories of age-associated changes of neuronal volume in the autistic cohort in comparison to controls

**Figure 5:**
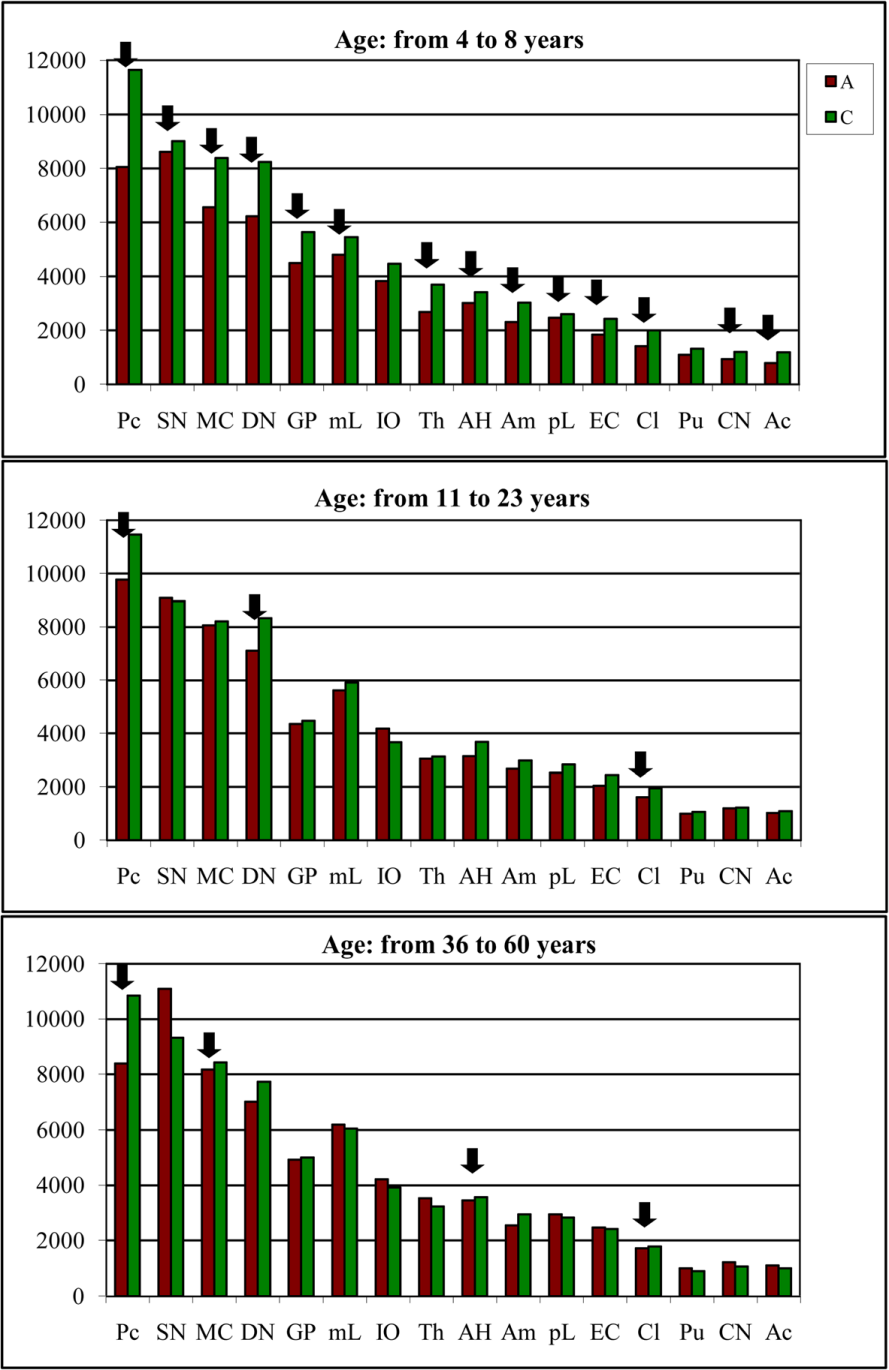
**Graphs demonstrate changes of the mean neuronal volume in three age groups of autistic and control individuals.** In from 4- to 8-year-old autistic subjects, the mean neuronal soma volume is significantly less (p < 0.05) in 14 of 16 examined brain regions (marked with black arrows) than in age-matched controls. The number of regions with significant deficit of neuronal soma volume decreases to three in autistic teenagers/young adults and to four regions in 36 to 60 years-old autistic individuals.

To detect the age at the major increase in the volume of neurons in the autistic subjects, the older than–8 years cohort was divided into two age groups, including six autistic subjects from 11 to 23 years of age and four from 36 to 60 years of age. Age-matched control groups consisted of four subjects from 14 to 23 years of age and six subjects from 29 to 64 years of age. The study revealed two region-specific trends with (a) persistent neuronal volume deficits in all three age groups and (b) increase of neuronal volume so close to age matched control subjects that the difference became insignificant. While in the 4- to 8-year-old autistic subjects significant volume deficits were present in 14 subregions, the comparison of neuronal volumes in the autistic and control subjects from 11 to 23 years revealed significant volume deficits in only 3 of 16 examined regions (Figure 5 and Additional file 1: Table S2). In the autistic subjects older than 36 years of age, significant neuronal volume deficits were present in four regions. This analysis identified the Purkinje cells and neurons in the claustrum as the only subregions with persistently lower neuronal soma volumes in the autistic subjects compared to controls during the entire lifespan. These data indicate that neuronal volumes in 81% of the structures in from 11 to 23-year-old autistic individuals and 75% of the structures in 36 to 60-year-old autistic subjects are indistinguishable from neuronal volumes of age-matched control subjects.

### Differences between the trajectory of neuronal volume changes within the autistic and control cohorts during the lifespan

**Figure 6:**
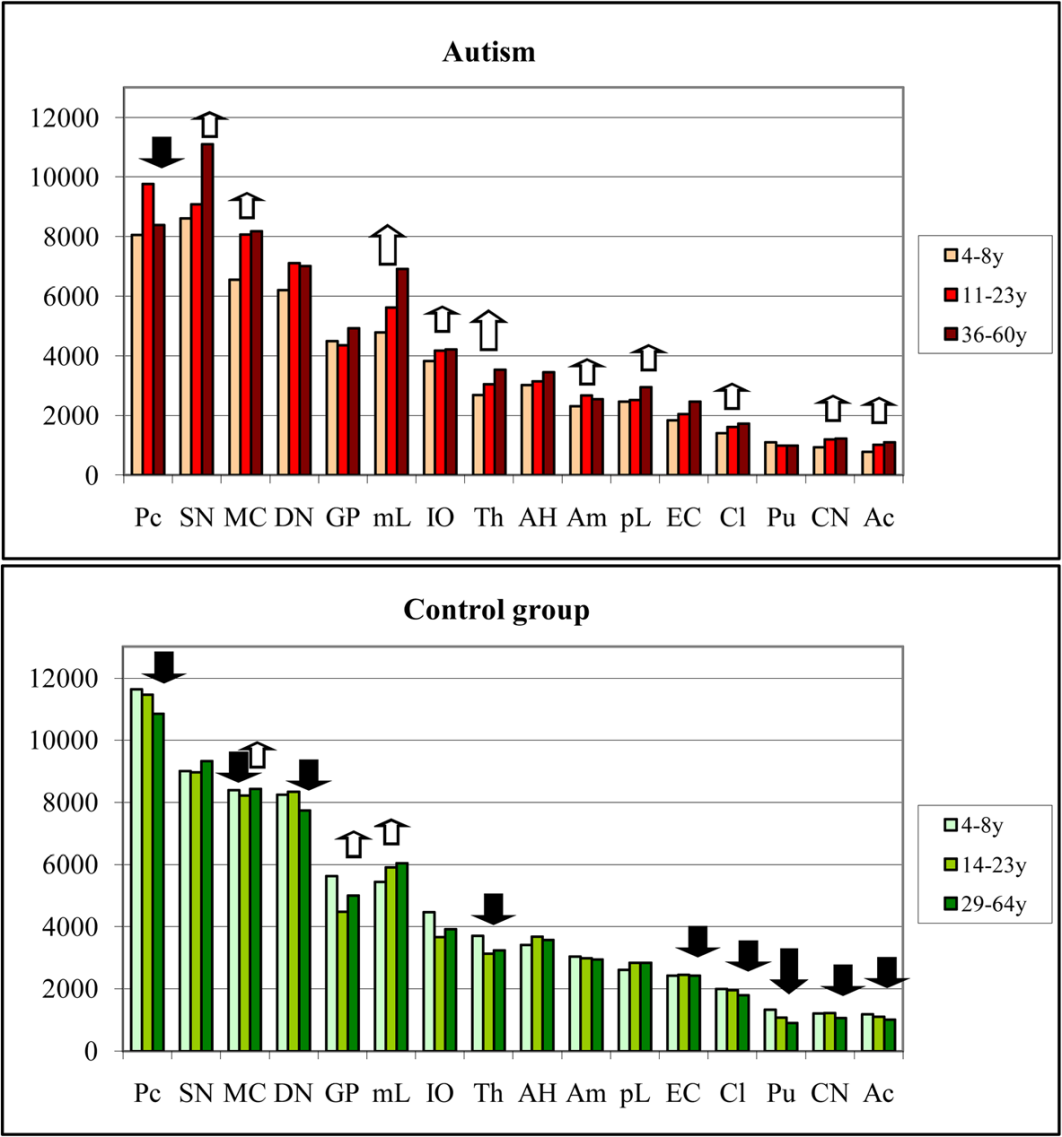
**Trends of the neuronal volume changes throughout lifespan.** In autistic teenagers/young adults and older adults neuronal soma volume increases in 10 regions (white arrows). The opposite trend is observed in the control group with neuron soma volume decreasing with age in nine examined structures (black arrows). Large arrows mark a significant increase or decrease of neuron volume in both older groups. Small arrows mark a significant increase or decrease in one of two older groups.

The first analysis characterized the relative age-associated changes of neuron soma volume in autistic subjects in comparison to controls. The aim of the second analysis was to characterize independently the trajectory of neuronal volume changes within the autistic cohort and within the control cohort. This analysis revealed opposite trends, with an increase in neuronal volumes in both autistic teenagers and adults, and a decrease of neuronal sizes in the majority of regions examined in both older control groups (Figure 6 and Additional file 1: Table S3).

In autistic teenagers/young adults (11–23 years of age), a significant increase (*p* < 0.02) in neuronal volumes (in comparison to the 4- to 8-year-old children with autism) was detected in eight regions, including the nucleus accumbens, 31%; claustrum 15%; thalamus, 14%; amygdala, 16%; magnocellular basal complex, 23%; caudate nucleus, 27%; inferior olive, 9%; and magnocellular LGB, 17%. Further significant increase of neuron soma volume was also observed in autistic subjects 36–60 years of age in the thalamus, 16%; magnocellular and parvocellular LGB, 23% and 17% respectively, and in the substantia nigra, 22%. In 36-60-year-old autistic adults reduced neuron soma volume (14%; *p* < 0.001) was detected only in Purkinje cells. The increase in neuronal soma volumes in 10 regions examined in one or both older groups defined the dominant feature of the trajectory of neuronal volume changes during the lifespan of autistic subjects.

The increase of neuronal volume in autistic cohort is in contrast with significant reduction of neuronal soma volume in nine regions in one or both older groups of control subjects. Significant decrease of neuron soma volume was detected in the 14- to 23-year-old control subjects in magnocellular basal complex, −2%; thalamus, −15%; and putamen, −19%. In control individuals from 29 to 64 years old a further significant decrease of neuron soma volume was detected in five regions, including the nucleus accumbens, −9%; Purkinje cells, −5%; claustrum, −8%; dentate nucleus, −7%; entorhinal cortex, −1%; caudate nucleus, −13%, and putamen, −16%. In control subjects the volume of neuronal soma increased in only three regions, including the magnocellular basal complex (2%), globus pallidus (12%) and magnocellular LGB (8%).

## Discussion

Results of this study support the hypotheses that (1) autism is associated with global developmental alterations of neuronal growth including subcortical structures, hippocampus, archicortex, cerebellum, and brainstem; (2) the trajectories of neuronal growth in children, teenagers/young adults, and adults diagnosed with autism, and age-matched control individuals are different, and that (3) the severity of neuronal growth abnormalities is region-specific.

### Difference between trajectories of neuronal growth in autistic and control subjects

This postmortem study was designed to detect developmental and age-associated modifications in the brain of autistic subjects. Neuronal soma volumes were selected as a technically, relatively precisely controlled marker of neuronal developmental and age-associated modifications both in the normal developing brain and in the brain with developmental abnormalities. That the most severe neuronal volume deficits were found in the 4- to 8-year-old children with autism suggests that a dysregulation of neuronal growth occurs before the age of four years. The significantly smaller volumes of neuronal soma in 14 brain structures involved in all the diagnostic autism domains suggest that these early alterations may contribute to the broad spectrum of clinical autism manifestations. The onset of the clinical features of autism is gradual in many children, but in other children, a functional regression has been reported in the first 1–2 years [65,66]. Some studies suggest a prenatal onset of developmental abnormalities leading to autism [67,68]. The pattern of changes seen in the cerebellum suggests that the pathology was acquired early in development [2]. This view is also supported by our findings of focal pre- and postnatal developmental defects, including abnormal migration with heterotopic and ectopic clusters of neurons, dysplastic changes, and subependymal nodular dysplasia detected in the brain of individuals with idiopathic and dup(15)-associated autism [5].

The greater neuronal volumes in eight brain regions of autistic subjects from 11 to 23 years old, and in four regions of autistic individuals from 36 to 60 years old, when compared to the 4 to 8 years old autistic subjects, is in striking contrast with decrease with age of neuronal volumes in nine regions of age-matched control subjects. Similar decreases in perikaryal volume of neurons in layer V in the fusiform gyrus have been reported in control subjects from 4 to 65 years of age [5]. A report by Jacot-Descombes et al. [4] also demonstrated a decrease in pyramidal neuronal volumes in BA 44 and 45 across the lifespan of control but not autistic subjects. Andersen et al. [69] detected in a control cohort an age-associated reduction of the size of the Purkinje cell perikaryon. A reduction of the size of perikarya was also detected in the external pyramidal layer in the superior part of the precentral gyrus but only in 85- to 94-year-old subjects, and these changes were interpreted as a sign of age-associated neuronal atrophy [70]. It is not known why the volume of cortical pyramidal neurons, Purkinje cells, and neurons in subcortical structures decreases in control subjects, but the absence of this decline in autistic subjects in some structures and the increase in neuronal size in other brain regions appear to be a marker of the abnormal trajectory of neuronal growth in teenagers and adults with autism.

### Global pattern of abnormal growth of neurons in the brain of autistic subjects and region-specific trajectories of neuronal growth

#### Small neurons in the neocortex of autistic individuals

Small sizes of neurons in autistic subjects have been reported in neuropathological evaluations without morphometric support [2,71-73], and in morphometric studies that estimate neuronal volume [4,5,18,19]. A small size of neurons has been reported in 22- to 29-year-old autistic subjects in the hippocampus, amygdala, medial septal nucleus, cerebellar nuclei, and inferior olive [2,71-73]. Casanova et al. [3] revealed reduced minicolumn widths, increased neuronal density, and reduced neuronal size in the superior and middle frontal gyrus in six autistic subjects 4–24 years of age compared to six age-matched control subjects. A study of seven autistic subjects, 4 to 23 years of age, and 10 control subjects, 4 to 65 years of age, revealed a reduced mean perikaryal volume of neurons in layers V by 21%, and VI by 13.4% in the fusiform gyrus. A reduced total neuronal number and smaller neurons in the main output layers of the fusiform gyrus were functionally linked to impaired face processing in autism [5]. A study of the inferior frontal cortex areas involved in language processing, imitative functioning, and social processing (Brodmann areas 44 and 45) revealed smaller pyramidal neuronal volumes in layers III, V, and VI in autistic subjects, 4 to 52 years old, compared to control subjects, 4 to 48 years of age [4]. A study of the anterior cingulate cortex in nine males with autism from 15 to 54 years of age revealed significant decreases in neuronal volumes in layers I–III and V–VI [19], suggesting a contribution to modified affective and cognitive behaviors, socio-emotional attachments, emotional self control, adaptive responses to changing conditions, goal-directed behavior, joint attention, and motor activity [74,75].

The majority of the brains of autistic subjects examined in four other morphometric studies [3-5,21] represent a portion of the cohort examined in this study. The reduced volume of neurons detected in 14 of 16 examined subcortical structures, archicortex, and cerebellum, combined with the results of the neocortical and brainstem studies of other investigators, strengthens the hypothesis of the global nature of defective neuronal growth in the brains of autistic subjects.

#### Amygdala, hippocampus, and entorhinal cortex

All individuals with autism display features of a deficit of social behavior, including abnormalities in social reciprocity and difficulties in the use of eye contact, facial expression, and social motivation [1]. Neurobiological models of social cognition suggest that in the neuronal network engaged in social cognition, including the amygdala, the cortex of the temporal superior sulcus, and the fusiform gyrus, it is the amygdala that is responsible for labeling events with emotional meaning [7,8]. Moreover, the amygdala plays a role in the detection of threats and the mobilization of an appropriate behavioral response including fear and anxiety [47]. Excessive anxiety is reportedly observed in 84% of children with autism [76]. Neuropathological data [72] as well as the results of structural [30,77] and functional neuroimaging [78] provide evidence that the amygdala is affected in autism and that pathology of the amygdala may contribute to the clinical deficits of autistic subjects. Our observed 24% volume deficit of neurons (*p* < 0.001) in the amygdala of the 4- to 8-year-old autistic subjects in comparison to control children suggests that during the most critical stage of development of social behaviors and emotional relationships, the growth of neurons in the amygdala is altered. The finding of a reduced size of neurons to a comparable range in the entorhinal cortex and in the amygdala (−24% in each; *p* < 0.001) in the 4- to 8-year-old children with autism, but of only a 12% neuronal volume deficit (*p* < 0.016) in the Ammon’s horn, suggests that the limbic system is developmentally affected, but the range of alterations in closely connected and interacting structures may be different.

#### Claustrum

Interconnectivity with subcortical nuclei and sensory cortical areas indicates the claustrum’s involvement in sensorimotor integration and potentially the most complex human brain function, consciousness, as well as in higher orders of functionality, enabling the organism to rapidly adapt to the subtleties and nuances of a changing environment [79,80]. It appears that developmental alterations of the claustrum, indicated in the neuronal soma deficit of 29% in children, of 17% in teenagers/young adults, and of only 4% in older adults (*p* < 0.001), reflect claustral neurons’ functional impairment contributing both to deficits of adaptability and consciousness and to a partially delayed reduction of the deficit with age similar to that seen in the limbic system.

The differences in the range of developmental delay of the claustral neurons and the neurons receiving claustral projections and projecting to the claustrum, suggest that claustral dysfunction is associated with or caused by desynchronized development of the subcomponents of these multifunctional networks. The claustrum is involved in long-term response potentiations within the claustral–entorhinal–hippocampal system [81] that is affected with neuronal volume deficits of 29%, 24% and 12%, respectively. Neurons in the frontal, temporal, parietal, and occipital cortices project to the claustrum [82,83], whereas neurons in the dorso-caudal claustrum (visual claustrum) project to the visual cortex. Only a slight reduction of neuronal volume in Brodmann area 17 in the occipital cortex [3] and a significant reduction in the mean perikaryal volume of the neurons in layers V and VI (by 21.1% and 13.4%, respectively) in the fusiform gyrus [5] are indicative of a desynchronized development of the claustro-cortical networks in autism.

#### Magnocellular basal complex (MBC)

The MBC consists of four major nuclei that send cholinergic, GABAergic, and glutamatergic axons to the cortical mantle, amygdala, and many subcortical structures [53,54], and its associated abnormalities may contribute to the clinical phenotype of autism. The cholinergic drive to the forebrain plays a modulatory role in anxiety, arousal, and attention and is essential for learning and memory tasks [15,16]. The anteromedial part of the MBC (CH4) acts as the cholinergic relay for transmitting limbic and paralimbic information to the neocortex, thereby influencing complex behavior (integrated emotional and motor responses, learning, and memory), according to the prevailing emotional and motivational states encoded by the limbic and paralimbic brain structures. Ch4 neurons respond to the sight and taste of food, visual and auditory information. All the structures that project to the Ch4 are integrative regions of extensive sensory processing or regions of polysensory convergence. The 22% neuronal soma volume deficits observed in MBC in 4- to 8-year-old autistic subjects may reflect a defective function of the cholinergic system in early childhood.

#### Thalamus

Experimental studies have shown that almost all amygdaloid nuclei project to the thalamus and that their main target is the rostral half of the magnocellular mediodorsal thalamic nucleus [84,85], which is involved in attention, emotional processing, anxiety, obsessive thinking, agitation, and assaultive behavior [9,10]. The lateral thalamus is closely related to language function, including the mechanical processes for articulation and respiration. The pallidal input to the thalamus serves to control muscle tone. MRI studies have revealed significantly reduced mean thalamic volume in high-functioning autistic subjects [86]. Our observations of much smaller neuronal soma (−27%; *p* < 0.001) in the thalamus of autistic subjects 4 to 8 years of age indicate that the neuronal circuits involved in articulation, attention, anxiety, and obsessive thinking belong to the most developmentally affected brain structures. The increase in neuronal soma volumes to control levels in teenagers and adults may reflect age-associated modifications of neuronal growth and possibly function.

#### Cerebellar cortex, dentate nucleus, and inferior olive

A reduced number and size of Purkinje cells was observed in the majority of cerebellar analyses in 21 of 29 examined cases [87]. Quantitative studies suggest that a regional decrease in the number of Purkinje cells may be the result of a prenatal loss of Purkinje cells [81,88]. Fatemi et al. [89] proposed that the 24% reduction in the cross-sectional areas of Purkinje cells that they detected in five young adults with autism (25 years of age on average) was a sign of neuronal atrophy. Our study revealed very severe Purkinje cell soma volume deficits (−31%; *p* < 0.001) in 4- to 8-year-old children with autism, and less severe deficit in 11- to 23-year-old (−15%; p <0.010) and more than 29 years of age (−23%; *p* < 0.001) autistic individuals. However, the significant decrease of neuron volume was observed when 11–23 y old and 36–60 y old autistic subjects were compared. This selective decrease only of Purkinje cell volume and only in the oldest autistic group may reflect combination of common metabolic alterations [90] and selective-age associated Purkinje cell atrophy [89].

These signs of developmental alterations coincide with a 40% decrease in the expression of glutamic acid decarboxylase 67 (GAD67) mRNA in autistic subjects [91] and an increased GABAergic feed-forward inhibition to Purkinje cells by basket cells, suggesting a disruption in the timing of Purkinje cell firing and altered inhibition of the cerebellar nuclei, which could directly affect cerebello-cortical output, leading to changes in motor behavior and cognition [92]. Our observed smaller volumes of Purkinje cells by 31%, of the dentate nucleus by 25%, and insignificant changes in the inferior olive (14%) in 4- to 8-year-old autistic subjects compared to age-matched controls, suggest brain region-specific range of alterations within different components of the brainstem-cerebellar circuits. A unique form of developmental disruption of brainstem/cerebellar networks is floccular dysplasia, which may contribute to the oculomotor system alterations resulting in avoidance of eye contact and poor or no eye contact observed in autistic subjects [93].

#### Nigro-striatal system

The substantia nigra, caudate nucleus, putamen, nucleus accumbens, and globus pallidus belong to the basal ganglia. They form cortico-subcortical circuits that play a key role in programming and execution of movements and in the reward system. An inverse correlation between the caudate nucleus volume and the presence of compulsions and rituals, and difficulty in changing routines was detected in 12- to 29-year-old autistic patients [94]. An increased volume of the caudate nuclei was proportional to compulsions and rituals [12]. The volume of the right caudate nucleus correlates with repetitive behaviors in 17- to 55-year-old ASD subjects [95]. Because of its anatomy and connectivity, the nucleus accumbens is considered a mixed structure with elements of the striatum and “extended amygdala” [96]. The nucleus accumbens is required for a number of reward-related behaviors, and it processes specific information about reward availability, value, and context [11]. Its projections to motor areas such as the ventral pallidum turn reward information into motivated action [97]. This study revealed significantly smaller (p < 0.001) volumes of neurons in the substantia nigra (−5%), caudate nucleus (−22%), globus pallidus (−20%), and nucleus accumbens (−34%), and non significant deficit (−17%) in the putamen of children with autism. These findings suggest that repetitive motor behaviors, circumscribed patterns of interests, rituals, and compulsions are the functional consequences of the abnormal development and maturation of nigro-striatal networks.

#### Closing remarks

Recent genetic findings along with anatomical and functional imaging studies suggest an ASD model in which higher-order association areas of the brain, which normally connect to the frontal lobe, are partially disconnected during development (“a developmental disconnection syndrome”) [98-100]. According to Geschwind and Levitt [100], the disconnection in ASDs is not primarily a disruption of previously connected regions but rather a failure of them to develop normally. This morphometric study of 16 brain regions and the results of neocortical studies demonstrate a failure of normal neuronal development that may contribute to a failure of normal connectivity development. Brain region–specific neuronal volume deficits and different trajectories of neuronal volume modifications during the lifespan indicate that an integral component of this developmental failure is the desynchronized growth of neurons, and that this might be a major structural contribution to the autism phenotype. The trajectory of neuronal growth detected in 4–8, 11–23, and 36–60 years of age autistic individuals resulting in an increase of neuronal soma volumes to those of control adults appears to reflect delayed up-regulation of neuron growth. The disproportion between (a) a strikingly strong trend to increase neuron soma volume close to control level in the majority of examined brain regions in autistic teenagers and adults, and (b) limited clinical improvement during autistic individual lifespan [35,37,38] suggests that mechanisms controlling delayed neuron growth do not replicate normal neuron development, maturation and connectivity. One may suspect that size normalization could be indicative of different processes, some of which may be deleterious due to the abnormal developmental timing.

## Competing interest

The authors declare that they have no competing interests.

## Supplementary Material

Additional file 1: Table S1 Parameters and procedures applied to estimate the volume of neuronal soma. **Table S2.** The difference between the mean volume of neuronal soma in autistic and control cohorts. **Table S3.** The trajectory of neuronal volume changes during lifespan of autistic and control subjects. Click here for file
