# Supplementary material for: Brain-region–specific alterations of the trajectories of neuronal volume growth throughout the lifespan in autism
Source: Acta Neuropathol Commun. 2014 Mar 10;2:28. doi: 10.1186/2051-5960-2-28 (PMC4007529; doi:10.1186/2051-5960-2-28)
Supplement: Additional file 1: Table S1 — Parameters and procedures applied to estimate the volume of neuronal soma. Table S2. The difference between the mean volume of neuronal soma in autistic and control cohorts. Table S3. The trajectory of neuronal volume changes during lifespan of autistic and control subjects. [file 2051-5960-2-28-S1.doc]

**Table S1** Parameters and procedures applied to estimate the volume of neuronal soma

| Structures  and their  subdivisions | Number  of equal  distance  sections  examined  (per case) | Obj. | Grid size  (μm) | Test area  frame  x height  (μm) | Mean  number  of virtual  counting  spaces  (per case)  ±SD | Mean  number  of neurons  measured  (per case)  ±SD | CE* |
| --- | --- | --- | --- | --- | --- | --- | --- |
| Amygdala | 12 | 40x | 1000x1000 | 80x80x10 | 356 ± 39 | 454 ± 90 | 0.01 |
| Thalamus | 6 | 40x | 1000x1000 | 80x80x10 | 188 ± 28 | 313 ± 59 | 0.01 |
| Entorhinal c. (LII-VI) | 6 | 40x | 400x400 | 60x60x30 | 355 ± 92 | 502 ±110 | 0.003 |
| CA1-4 | 14 | 40x | 400x400 | 60x60x30 | 232 ± 54 | 509 ± 60 | 0.003 |
| Caudate nucleus | 4 | 63x | 2000x2000 | 80x80x30 | 74 ± 21 | 244 ± 80 | 0.002 |
| Putamen | 4 | 63x | 2000x2000 | 80x80x30 | 73 ± 21 | 255 ± 80 |
| Globus pallidus | 4 | 63x | 500x500 | 180x180x30 | 117 ± 48 | 137 ± 52 |
| Nucleus accumbens | 4 | 63x | 500x500 | 180x180x30 | 134 ± 47 | 353 ± 59 |
| Magnocellular LGB | 4 | 40x | 500x500 | 100x100x30 | 114 ± 61 | 190 ± 6 | 0.002 |
| Parvocellular LGB | 4 | 40x | 500x500 | 100x100x30 | 123 ± 62 | 211 ± 20 | 0.002 |
| Claustrum | 9 | 40x | 250x250 | 60x60x10 | 274 ± 96 | 318 ± 48 | 0.01 |
| Substantia nigra | 9 | 63x | 300x300 | 80x80x30 | 108 ± 31 | 287 ± 64 | 0.002 |
| Magnocellular  basal complex  (Ch1-Ch4) | 9 | 63x | 300x300 | 80x80x30 | 59 ± 22 | 153 ± 44 | 0.005 |
| Purkinje cells | 4 | 40x | 1800x1800 | 180x180x30 | 664 ±302 | 253 ± 40 | 0.002 |
| Dentate nucleus | 4 | 40x | 1000x1000 | 180x180x30 | 145 ± 37 | 254 ± 56 | 0.002 |
| Inferior olive | 4 | 40x | 1000x1000 | 180x180x30 | 132 ± 41 | 260 ± 62 | 0.002 |

Obj., objective; *CE, the average predicted coefficient of error of the measured neurons (Scheaffer); LGB, lateral geniculate body.

**Table S2** The difference between the mean volume of neuronal soma in autistic and control cohorts

| Brain  structure | 4- to 8-year-old subjects | | 11- to 23-year-old subjects | | 29- to 64-year-old subjects | |
| --- | --- | --- | --- | --- | --- | --- |
|  | Control | Autism | Control | Autism | Control | Autism |
|  | Mean (LSE)  (100%) | Mean (LSE)  *p* < (%) | Mean (LSE)  (100%) | Mean (LSE)  *p* < (%) | Mean (LSE)  (100%) | Mean (LSE)  *p* < (%) |
| Nucleus  accumbens | 1,181 (9) | 1. (7)   0.001 -34% | 1,090 (8) | 1,021 (7)  0.285 ns | 997 (7) | 1,102 (8)  0.926 ns |
| Cerebellum.  Purkinje cells | 11,635 (104) | 8,047 (97)  0.001 -31% | 11,460 (112) | 9,774 (80)  0.010 -15% | 10,845 (82) | 8,389 (85)  0.001 -23% |
| Claustrum | 1,994 (14) | 1,407 (13)  0.001 -29% | 1,950 (14) | 1,614 (11)  0.001 -17% | 1,785 (13) | 1,722 (19)  0.001 -4% |
| Thalamus | 3,702 (36) | 2,682 (35)  0.001 -27% | 3,135 (37) | 3,051 (30)  0.979 ns | 3,230 (31) | 3,535 (39)  0.192 ns |
| Dentate nucleus | 8,243 (153) | 6,218 (121)  0.001 -25% | 8,331 (158) | 7,106 (103)  0.019 -15% | 7,732 (112) | 7,006 (124)  0.288 ns |
| Amygdala | 3,033 (28) | 2,309 (26)  0.001 -24% | 2,982 (30) | 2,674 (22)  0.034 ns | 2,946 (27) | 2,552 (27)  0.069 ns |
| Entorhinal  cortex | 2,424 (29) | 1,841 (23)  0.001 -24% | 2,450 (37) | 2,041 (23)  0.895 ns | 2,420 (23) | 2,464 (27)  0.097 ns |
| Magnocellular  basal complex | 8,385 (67) | 6,553 (56)  0.001 -22% | 8,214 (74) | 8,063 (66)  0.434 ns | 8,425 (83) | 8,174 (126)  0.001 -3% |
| Caudate  nucleus | 1,199 (13) | 936 (12)  0.001 -22% | 1,218 (12) | 1,192 (10)  0.115 ns | 1,056 (10) | 1,217 (11)  0.148 ns |
| Globus  pallidus | 5,636 (70) | 4,502 (74)  0.001 -20% | 4,478 (70) | 4,354 (52)  0.210 ns | 5,001 (56) | 4,930 (59)  0.549 ns |
| Putamen | 1,316 (12) | 1,095 (12)  0.057 ns | 1,065 (11) | 988 (8)  0.162 ns | 891 (8) | 993 (9)  0.526 ns |
| Inferior  olive | 4,465 (76) | 3,833 (54)  0.330 ns | 3,661 (63) | 4,172 (43)  0.665 ns | 3,917 (42) | 4,219 (85)  0.455 ns |
| Ammon’s  horn | 3,414 (31) | 3,020 (28)  0.016 -12% | 3,675 (32) | 3,147 (25)  0.052 ns | 3,575 (27) | 3,459 (29)  0.001 -3% |
| Magnocellular  LGB | 5,448 (77) | 4,795 (68)  0.001 -12% | 5,908 (87) | 5,622 (64)  0.320 ns | 6,046 (75) | 6,197 (77)  0.702 ns |
| Parvocellular  LGB | 2,605 (31) | 2,466 (30)  0.001 -5% | 2,828 (34) | 2,524 (25)  0.101 ns | 2,831 (30) | 2,949 (31)  0.543 ns |
| Substantia  nigra | 9,008 (65) | 8,604 (61)  0.001 -5% | 8,957 (65) | 9,087 (51)  0.077 ns | 9,324 (71) | 11,101 (116)  0.153 ns |

LSE, linearized standard error; n, LGB, lateral geniculate body. Significance levels computed controlling for post-mortem interval (log-transformed), days of dehydration, and weight loss during dehydration.

**Table S3** The trajectory of neuronal volume changes during lifespan of autistic and control subjects

| Brain  structure | Autism | | | | | Control | | | | |
| --- | --- | --- | --- | --- | --- | --- | --- | --- | --- | --- |
|  | A | B | C | Diff % | Diff % | A | B | C | Diff % | Diff % |
|  | 4-8 y | 11-23 y | 36-60y | A/B | B/C | 4-8 y | 14-23 y | 29-64 y | A/B | B/C |
| Nucleus  accumbens | 779 | 1,021 | 1,102 | 31%  0.001 | 8%  ns | 1,181 | 1,090 | 997 | - 8%  ns | -9%  0.001 |
| Cerebellum.  Purkinje cells | 8,047 | 9,774 | 8,389 | 21%  ns | -14%  0.001 | 11,635 | 11,460 | 10,845 | -2%  ns | -5%  0.004 |
| Claustrum | 1,407 | 1,614 | 1,722 | 15%  0.003 | 7%  ns* | 1,994 | 1,950 | 1,785 | -2%  ns | -8%  0.001 |
| Thalamus | 2,682 | 3,051 | 3,535 | 14%  0.010 | 16%  0.025 | 3,702 | 3,135 | 3,230 | -15%  0.001 | 3%  ns |
| Dentate nucleus | 6,218 | 7,106 | 7,006 | 14%  ns | -4%  ns | 8,243 | 8,331 | 7,732 | 1%  ns | -7%  0.025 |
| Amygdala | 2,309 | 2,674 | 2,552 | 16%  0.001 | -5%  ns | 3,033 | 2,982 | 2,946 | -2%  ns | -1%  ns |
| Entorhinal  cortex | 1,841 | 2,041 | 2,464 | 11%  ns | 21%  ns | 2,424 | 2,450 | 2,420 | 1%  ns | -1%  0.001 |
| Magnocellular  basal complex | 6,553 | 8,063 | 8,174 | 23%  0.001 | 1%  ns | 8,385 | 8,214 | 8,425 | -2%  0.001* | 2%  0.001 |
| Caudate  nucleus | 936 | 1,192 | 1,217 | 27%  0.001 | 2%  ns | 1,199 | 1,218 | 1,056 | 2%  ns | -13%  0.001 |
| Globus  pallidus | 4,502 | 4,354 | 4,930 | -3%  ns | 13%  ns | 5,636 | 4,478 | 5,001 | -21%  ns | 12%  0.007 |
| Putamen | 1,095 | 988 | 993 | -10%  ns | 0%  ns | 1,316 | 1,065 | 891 | -19%  0.014 | -16%  0.001 |
| Inferior  olive | 3,833 | 4,172 | 4,219 | 9%  0.017 | 1%  ns | 4,465 | 3,661 | 3,917 | -18%  ns* | 7%  ns |
| Ammon’s  horn | 3,020 | 3,147 | 3,459 | 4%  ns | 10%  ns | 3,414 | 3,675 | 3,575 | 8%  ns | -3%  ns |
| Magnocellular  LGB | 4,795 | 5,622 | 6,917 | 17%  0.002 | 23%  0.001 | 5,448 | 5,908 | 6,046 | 8%  0.001 | 2%  ns |
| Parvocellular  LGB | 2,466 | 2,524 | 2,949 | 2%  ns | 17%  0.001 | 2,605 | 2,828 | 2,831 | 9%  ns | 0%  ns |
| Substantia  nigra | 8,604 | 9,087 | 11,101 | 6%  ns | 22%  0.001 | 9,008 | 8,957 | 9,324 | -1%  ns | 4%  ns |

Significance levels of the differences in mean volumes between age groups (Diff %), were computed controlling for post-mortem interval (PMI; log-transformed), days of dehydration, and weight loss. Results marked “ns” were non-significant using a significance criterion of p < 0.05.

*PMI omitted from model to avoid overfitting and/or multicollinearity.

The apparent inconsistencies between the percentage differences observed and the calculated significance levels are due in large part to the controlling of potential confounders.
